# To Prevent Beds Damaging Walls

**Published:** 1913-05-10

**Authors:** Helen M. Brown

**Affiliations:** (Matron). Daneswood Sanatorium, Woburn Sands, Beds


*To the Editor of* The Hospital.

### To Prevent Beds Damaging Walls.

*To the Editor of* The Hospital.

Sir,—A correspondent of yours in this week's Hospital is inquiring for a contrivance to prevent bedsteads from damaging walls in institutions, and I would draw her attention to the fact that owing to our own walls here suffering very much from the same cause I have just lately patented a device for the purpose. It has been taken up by the "Lawson Tait" bedstead makers, and is very shortly to be put on the market. It is a metal and rubber contrivance, and is easily affixed to the leg or the rail of the bed. Projecting as it does beyond the most prominent portion of the bed, it effectually acts as a buffer. The rubber portion is revolving, and so the bed can be pushed along the wall without any damage being done.

Whitfield's Bedstead Company, of Birmingham, are the sole makers, and will supply all information.—I am, dear Sir, yours faithfully,

Helen M. Brown (Matron).

Daneswood Sanatorium, Woburn Sands, Beds,
May 2.

